# MELACARE Nurse-led follow-up after early-stage melanoma: protocol and feasibility

**DOI:** 10.2340/1651-226X.2024.41037

**Published:** 2024-11-24

**Authors:** Sara M. Hansen, Christoffer Johansen, Magnus P.B. Obinah, Nadine A. Kasparian, Peter Genter, Pernille E. Bidstrup, Lisbet R. Hölmich

**Affiliations:** a Department of Plastic Surgery, Copenhagen University Hospital, Herlev and Gentofte, Herlev, Denmark; b Department of Oncology, Cancer Survivorship and Late Effects Research Center (CASTLE), Copenhagen University Hospital, Rigshospitalet, Copenhagen, Denmark; c Cincinnati Children’s Hospital Medical Center and Department of Pediatrics, Heart and Mind Wellbeing Center, Heart Institute and Division of Behavioral Medicine and Clinical Psychology, University of Cincinnati College of Medicine, Cincinnati, Ohio, United States; d Center for Mental Health, Copenhagen, Denmark; e Psychological Aspects of Cancer, Danish Cancer Institute, Copenhagen, Denmark; f Institute of Psychology, University of Copenhagen, Copenhagen, Denmark; g Department of Clinical Medicine, University of Copenhagen, Copenhagen, Denmark

**Keywords:** Cancer, fear of cancer recurrence, skin self-examination, patient-reported outcome, randomised controlled trial, intervention

## Abstract

**Background and purpose:**

We developed the Melacare nurse-led intervention, which combines education in skin self-examination as a resource-conscious approach to detecting recurrence and management of fear of cancer recurrence in patients treated for melanoma. This publication presents the Melacare study protocol and evaluates the feasibility and acceptability of Melacare prior to a larger randomised controlled trial.

**Material and methods:**

Feasibility and acceptability of Melacare were evaluated in an intervention-only feasibility study, in which patients attended two nurse-led intervention sessions coupled with an educational booklet. Participants completed patient-reported outcome (PRO) questionnaires at baseline and before each session. After the intervention, participants completed a study-specific feedback questionnaire. Feasibility was evaluated in terms of recruitment, adherence, and attendance. Self-reported outcomes from the study-specific questionnaire on intervention effects were also collected.

**Results of the feasibility study:**

Fourteen patients (nine stage IA, five stage IB melanoma) participated. Attendance and recruitment rates were 100%, all participants completed the baseline and PRO questionnaires, and 100% read at least half of the educational booklet. In terms of intervention effects, all patients reported improved knowledge of performing skin self-examination and coping with the fear of cancer recurrence.

**Interpretation:**

Results indicate that the Melacare nurse-led intervention is highly feasible and acceptable for use with patients treated for early-stage melanoma. Prior to clinical trial commencement, minor refinements include changing the method of recruiting by telephone and offering both in-person and telephone/video consultations to accommodate participant preference.

## Graphical abstract

**Figure:**
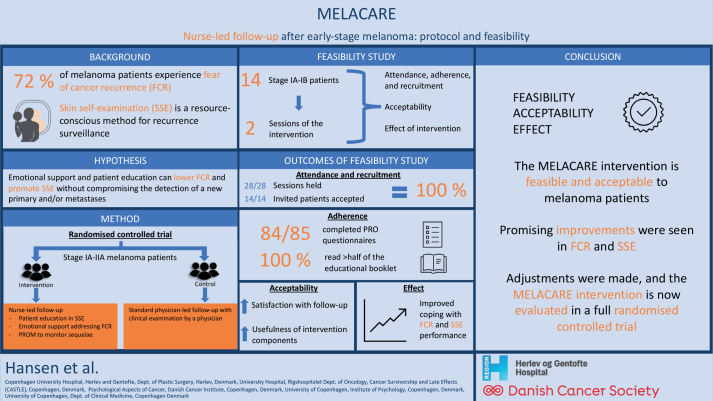

## Introduction

International guidelines on follow-up after surgery for cutaneous melanoma (melanoma) support a risk-based stratification to detect recurrence [1–3] and manage psychological sequelae [4]. The potential of combining education of patients in skin self-examination (SSE) as a resource-conscious approach to detect recurrence with management of psychological sequelae is unexplored and, to the best of our knowledge, has never been tested in a randomised controlled trial (RCT).

The Dutch MELFO RCT showed that a reduced-frequency follow-up was a safe and cost-effective alternative in 180 IB-IIC patients [5]. An American RCT involving 494 participants found that structured SSE training increased the reliability of self-reported SSE [6].

It has been shown that SSE may trigger the fear of cancer recurrence (FCR) [7], and it is therefore essential to evaluate if an FCR increase may be averted through psychosocial support. Up to 72% experience FCR, making this the most common psychological concern among individuals with melanoma [8, 9]. An Australian RCT including 183 individuals with a history of stages 0–II melanoma found that psychological support as part of standard care significantly reduced FCR at 6-month [10] and 12-month [11]. In addition, a Czech systematic review including 13 RCTs targeting different cancer types found that cognitive-behavioural therapy reduced FCR [12].

Building on the most promising intervention components to optimise recurrence detection [5, 6] and reduce FCR [10] among individuals with a previous melanoma diagnosis, we developed the Danish melanoma care study (Melacare) for patients treated for early-stage melanoma. The Melacare protocol and the intervention’s feasibility and acceptability are outlined further in the text. We hypothesise that Melacare may improve SSE for detecting new melanomas or recurrences and reduce FCR.

## Material and methods

### Study design

**Figure 1:**
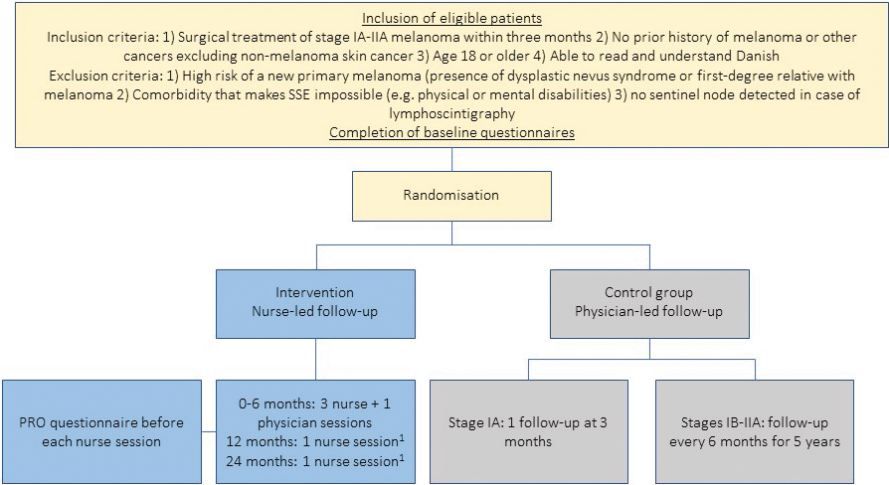
Study flow. ^1^Optional nurse sessions. PRO: patient-reported outcome; SSE: skin self-examination; Stage IA: IB-IIA refers to the American Joint Committee on Cancer, 8^th^ edition melanoma classification.

Melacare is a two-group RCT where patients randomised to intervention receive nurse-led follow-up, including education in SSE and FCR management, and patients randomised to control receive standard physician-led follow-up (Figure 1). The study is conducted at the Department of Plastic Surgery, Herlev and Gentofte Hospital, Copenhagen, Denmark, following SPIRIT and CONSORT recommendations [13].

### Participants and recruitment

Melacare includes patients who meet the inclusion and not the exclusion criteria (Figure 1). A study group member (medical doctor, medical student, or project nurse) invites eligible patients to participate by telephone. Included patients must provide written informed consent.

### Randomisation

The principal investigator randomly assigns patients 1:1 to the intervention or control group. Randomisation is computer-generated via the closed site www.melanoma.dk and stratified according to clinical stage. The principal investigator, melanoma nurses, and patients are not blinded to randomisation. The analyst will be blinded to conditions.

### The Melacare intervention

Melacare was designed to improve SSE skills relevant to recurrence detection and reduce FCR. The intervention includes:

1. Nurse-led follow-up, including three to five sessions with a melanoma nurse (Supplementary Appendix 1). SSE education is provided through in-person education, an introduction to the ABCDE rule [14], a step-by-step booklet, and guidance on symptoms requiring attention. FCR management is based on the Australian melanoma care intervention, which included three sessions delivered by psychologists to patients at high risk of a new melanoma [10]. Detached mindfulness (DM) and worry postponement (WP) are key tools applied to address FCR [15–18]; see Supplementary Appendix 1. The nurse sessions are supported by:
  - An educational booklet (‘Melanoma – Questions and Answers’), initially developed for the Australian intervention to provide written psychoeducation [19].
  - Melanoma-specific health-related quality of life assessment using the validated ‘Functional Assessment of Cancer Treatment – Melanoma’ (FACT-M) scale [20, 21] at each nurse session to address emotional and physical sequelae and refer patients to relevant treatment in case of alarm symptoms [4].
2. One scheduled consultation with a physician (plastic surgeon; resident or consultant) to demonstrate SSE and ensure adherence to the protocol (Supplementary Appendix 1). Patients may request additional consultations if they experience alarm symptoms indicating recurrence or a new primary melanoma.

### The control group

Patients in the control group receive standard physician-led follow-up visits with clinical examination. Stage IA patients have one follow-up visit after 3 months, and stage IB and IIA patients have one follow-up every 6 months for 5 years [4].

### Development of the Melacare intervention

The Melacare intervention was developed with the Australian study’s principal investigator (NK) [10, 16]. Material from the Australian melanoma care study included a 114-page manual for healthcare professionals and a 76-page booklet for patients; SMH translated both and, in collaboration with study group members, adjusted them to fit the current study context (e.g. the intervention is delivered by nurses). Psychologists delivered the Australian melanoma care intervention, which we adjusted to fit the competencies of routine melanoma nurses. A 3-day mandatory training program was developed for the nurses (Supplementary Appendix 2). A psychologist supervises the nurses during the first year of the study period. During the RCT, the in-person nurse sessions are recorded to evaluate fidelity.

### Outcome measures

The primary outcome is the between-group difference in FCR using the Danish version of the four-item Concerns About Recurrence Questionnaire 4 (CARQ-4) [22] at 6 months (Table 1). A higher CARQ-4 score indicates a greater FCR, with scores > 11 indicative of clinical FCR. Secondary outcomes are collected using validated self-report measures, study-specific items, and medical records (Table 1).

**Table 1:** Primary and secondary outcome measures and their timing.

Primary/secondary	Outcome	Measure	Items	Baseline	6 months	12 months	24 months	60 months
**Primary outcome (self-report)**	Fear of cancer recurrence	Concerns About Cancer Recurrence (CARQ-4) [22]	4	x	Primary endpoint	x	x	
**Secondary outcomes (self-report)**	Distress	Distress thermometer [23, 24]	2	x	x	x	x	
	Anxiety	General Anxiety Disorder-7 (GAD-7) [25, 26]	7	x	x	x	x	
	Depression	The Patient Health Questionnaire (PHQ-9) [27, 28])	9	x	x	x	x	
	Health status	EQ-5D-3L [29]	5	x	x	x	x	
	Activation	Patient Activation Measure (PAM) [30])	13	x	x	x	x	
	Workability	Work Ability Index (WAI)^a^ [31]	7	x	x	x	x	
	Reporting of skin self-examinations, used tools, and use of booklet^b^	Study-specific questionnaire	4	x	x	x	x	
	Satisfaction with the program	Study-specific questionnaire	1	x	x	x	x	
	Time spent getting to/and from the follow-up visits	Study-specific questionnaire	1					x
	Costs spend getting to/and from the follow-up visits	Study-specific questionnaire	2					x
**Secondary outcomes (registers/electronic medical records)**	Use of health care services related to melanoma^c^				x	x	x	x
	Number of excisions/biopsies of suspected nevi, recurrences, or metastasis				x	x	x	x
	Number and characteristics of new primary detected							x
	Number and characteristics of recurrence detected							x
	Time to diagnosis of a new primary							x
	Time to diagnosis of a recurrence							x
	Health care costs							x

a Excluding patients who have retired;

b Only patients in the intervention group;

c Extra scans, clinical examinations, excisions of suspected lesions.

### Patient characteristics

At baseline, self-reported marital status, education, number of children, and household income are obtained, and age, gender, and disease stage are obtained from the medical records.

### Sample size and power calculations

A total sample of 378 patients will be recruited. An expected 10% attrition will result in 340 patients, with 170 patients in each group. We expect an effect size Cohen’s *D* of at least 0.3, as found in a similar study at 6-month follow-up [10]. We will have a power of 79% to detect a small effect size of Cohen’s *D* = 0.3 and a power of 99.5% to detect a moderate effect size of Cohen’s *D* = 0.5. All calculations assume a significance level of 5% two-sided.

### Statistical analyses

In intention-to-treat analysis, the linear mixed model is employed to investigate whether the Melacare intervention can reduce FCR at 6 months. The linear model is adjusted for baseline FCR, constraining the mean in the two groups to be identical at baseline [32]. A random intercept is included for each individual. In secondary publications, linear mixed-effect models are applied to investigate the effect of the intervention on secondary outcomes at 12, 24, and 60 months. These models will similarly constrain the baseline means to be equal. The variance-covariance structure is modelled using a random intercept model unless alternative structures, such as AR [1] or unstructured variance-covariance, provide a better fit according to the Akaike Information Criterion (AIC). All models are adjusted for randomisation strata. If between-group differences at baseline are detected, additional adjustments for the relevant factors are made. If the distribution of the outcomes is affected by floor or ceiling effects, the outcomes will be normalised [33]. Survival analyses are applied to examine intervention effects on time to new melanoma and recurrences with 60-month follow-up. Explorative analyses are performed to examine if the intervention effect differs according to age, gender, marital status, and level of FCR at baseline. If missing data at follow-up exceeds 10%, models are adjusted for covariates predictive for missingness, assuming data is Missing at Random (MAR). A significance level of 0.05 is applied. R version 4.3.3 will be used for data analyses.

## Feasibility study of the Melacare intervention

We conducted an intervention-only feasibility study to examine feasibility, acceptability, and effect to evaluate whether we needed to adjust the study procedure or intervention content. Patients in the feasibility study participated in Sessions 1 and 2 of the Melacare intervention alongside their regular physician follow-up. Eligible patients who participated in a patient information meeting on melanoma were invited to participate. Inclusion and exclusion criteria were the same as for the planned RCT.

## Feasibility and acceptability measures

Baseline and patient-reported outcome (PRO) questionnaires were the same as in the RCT. Intervention feasibility was assessed after Session 2 in terms of recruitment (recruitment rate and method), adherence (questionnaire completion and booklet use), attendance (number of completed sessions), and feasibility-study-specific feedback questions on acceptability and effects (Supplementary Appendix 3). All analyses of the feasibility study are descriptive. A focus group interview evaluated the melanoma nurses’ acceptability.

## Feasibility study results

Of 37 eligible patients attending the patient information meeting, 14 random patients were invited to participate, and all accepted. Participant characteristics are summarised in Supplementary Appendix 4.

A total of 28 sessions were delivered with 100% attendance. All participants would be comfortable if Session 2 were delivered via telehealth. Questionnaire completion was 100% at baseline and for FACT-M. All participants read at least half of the booklet.

Most participants found the content useful, would participate in an RCT testing the present intervention if offered, and reported improved coping with FCR and SSE performance (Table 2). The melanoma nurses reported feeling confident and having enough time to deliver the intervention.

**Table 2:** Acceptability of Melacare.

Number of patients reporting the usefulness of	Not at all useful	Partly useful	Useful	Very useful
1st session	0	0	3	10
2nd session	0	0	4	9
ABCDE^a^ rule	0	0	5	8
Education in skin self-examination	0	1	5	7
Detached mindfulness	1	3	7	2
Worry postponement	1	2	7	3
	**Not at all satisfied**	**Partly satisfied**	**Satisfied**	**Very satisfied**
Satisfaction with the follow-up method	0	0	1	13
Would participate in an RCT, including this intervention				
Yes	13			
No	0			

a ABCDE rule refers to a set of guidelines to spot changes in the skin [14].

FACT-M: Functional Assessment of Cancer Therapy-Melanoma questionnaire; RCT: Randomised controlled trial.

## Discussion

We developed the Melacare intervention to teach effective SSE as a resource-conscious approach to recurrence detection and to reduce FCR in patients with early-stage melanoma. The feasibility study results show that the Melacare intervention was feasible and acceptable among these patients.

We recruited patients for the feasibility study at a patient information meeting, but the physical circumstances were not optimal, so the recruitment method will be via telephone for the RCT. The participation rate of invited patients in our feasibility study was higher than in a similar feasibility study of a nurse-led follow-up in breast cancer patients [34] (100% vs. 78%). It could be ascribed to the fact that the participants in our study also attended their regular physician follow-up alongside the nurse sessions of the intervention and, therefore, were not afraid of missing out on something. Attendance and adherence were excellent, indicating that the intervention format was appropriate. Also, the high completion rate of the PRO questionnaire before the nurse sessions demonstrates the feasibility of using PROs in a follow-up setting. Most participants found the Melacare intervention and its content acceptable and would participate in the RCT, emphasising the relevance of the intervention in this population. All participants experienced improved coping with FCR and SSE performance, which are the main aspects we want to address with the Melacare intervention.

The feasibility study’s strength is the excellent adherence and attendance; however, limitations include lack of fidelity evaluation and the fact that the setting did not completely reflect the RCT. Due to patient feedback, we changed the recruitment method and made Session 2 available as a telephone/video consultation.

## Conclusion

We developed the Melacare follow-up intervention protocol for early-stage melanoma patients to improve recurrence detection via SSE and reduce psychological sequelae, especially FCR, following melanoma diagnosis. The intervention was evaluated in a feasibility study, which supported the intervention components and study procedures. The feasibility study led to minor refinements. Based on these experiences, we initiated the RCT in 2021 and it is ongoing (ClinicalTrials.gov ID: NCT05253872). Data collection will continue, with follow-up ending in 2028.

## Supplementary Material

MELACARE Nurse-led follow-up after early-stage melanoma: protocol and feasibility

## Data Availability

According to Danish legislation, making patient data available to other parties is illegal, and therefore, feasibility data is only available as presented in this paper.
