# Supplementary material for: MELACARE Nurse-led follow-up after early-stage melanoma: protocol and feasibility
Source: Acta Oncol. 2024 Nov 24;63:41037. doi: 10.2340/1651-226X.2024.41037 (PMC11609874; doi:10.2340/1651-226X.2024.41037)
Supplement: MELACARE Nurse-led follow-up after early-stage melanoma: protocol and feasibility [file AO-63-41037-s1.pdf]

Supplementary material has been published as submitted. It has not been copyedited, or typeset by Acta Oncologica

Appendix 1. Content of sessions in the Melacare intervention

	Session	Content	Duration (minutes)	In-person, telephone or video consultation
	At all nurse sessions, the melanoma nurses empathetically listen to patients, address symptoms and concerns about recurrence and skin self-examination (SSE), and identify patterns needing support. They also assist with meta-cognitive skills and self-management and guide patients on when to seek further support.<br>Skills applied:<br>1) Detached mindfulness (DM): observing thoughts and feelings without fixation or reaction, seeing them as separate from the self. It aims to reduce worry, improve attention control, and lessen thoughts' influence on self-identity.<br>2) Worry postponement (WP): recording worries and delaying them to a set time, reducing their significance. This helps challenge beliefs about uncontrollable worry and often makes revisiting them unnecessary.			
Within 6 months of randomisation	1	Nurse session<br>• Exploration of the patient's melanoma story<br>• FACT-M questionnaire<br>• Training of SSE skills and introducing the ABCDE rule[a]<br>• Provision of booklet	60	In-person
		Physician consultation:<br>• Evaluation of SSE skills<br>• Demonstration of how to perform SSE	15	In-person
	2	Nurse session:<br>• Follow-up from last session<br>• FACT-M questionnaire<br>• Exploration of emotions, thoughts, and behaviours related to melanoma, FCR, and SSE<br>• Attention to patient's coping strategies and introduction to DM and WP	60	In-person or video

		• Repetition of SSE skills and the ABCDE rule		
	3	Nurse session:<br>• Follow-up from the last session hereunder the use of DM and WP<br>• FACT-M questionnaire<br>• Repetition of SSE skills and the ABCDE rule	30	Telephone or video
After 12 months	4	Nurse session:<br>• Follow-up from last session<br>• FACT-M questionnaire<br>• Repetition of SSE skills and the ABCDE rule	20	Telephone
After 24 months	5	Nurse session:<br>• Follow-up from last session<br>• Review FACT-M questionnaire<br>• Repetition of SSE skills and ABCDE rule	20	Telephone

[a] The ABCDE rule refers to a set of guidelines to spot changes in the skin (19)

FACT-M: Functional Assessment of Cancer Therapy – Melanoma, FCR: Fear of Cancer Recurrence

Appendix 2. Melanoma nurse training program

Topic	Lessons (45 min)
Study protocol	2
Nurse manual	3
Melanoma, detection of recurrence, and follow-up	2
Skin self-examination	2
FACT-M and flowcharts for actions	2
Fear of cancer recurrence	1
Empathic listening	3
Working with worries and use of Worry postponement	3
Detached mindfulness practice	3

FACT-M: Functional Assessment of Cancer Therapy – Melanoma

Appendix 3. Feedback questionnaire for participants in the Feasibility study of Melacare

<u>The following questions pertain to the first session:</u>

1. How useful was the 1st session with the nurse for your follow-up process after melanoma?

Not useful at all ☐ Partly useful ☐ Useful ☐ Very useful ☐

2. The purpose of the first session was for the nurse to gain an understanding of you and your melanoma journey. Do you feel that this purpose was fulfilled?

Yes ☐ No ☐

3. You were trained in self-examination your skin. Do you feel that you were adequately trained in self-monitoring your skin?

Yes ☐ No ☐

4. You were trained in the "ABCDE rule," which is a guideline for what to watch for on your skin. Do you feel that you were adequately trained in the ABCDE rule for your skin?

Yes ☐ No ☐

5. Due to the COVID-19 pandemic and feedback about parking issues and other practical challenges in getting to the hospital, we have considered whether the consultations could be either video or phone consultations. Do you feel the first session would work as a video consultation or phone call? You may check both "video consultation" and "phone call."

Video consultation ☐ Phone call ☐ Would only work as an in-person meeting at the clinic ☐

6. If you had been offered the choice of either a video consultation, phone call, or in-person meeting at the clinic for the 1st consultation, which option would you have chosen?

Video consultation ☐ Phone call ☐ In-person meeting at the clinic ☐

The following questions pertain to the second session:

1. How useful was the second session with the nurse for your follow-up process after melanoma?

Not useful at all ☐ Partly useful ☐ Useful ☐ Very useful ☐

2. The purpose of the second consultation was to address fears and concerns regarding your melanoma diagnosis and how it affects your life. Do you feel that this purpose was fulfilled?

Yes ☐ No ☐

3. You were introduced to a tool called "observing mindfulness." Do you feel that you were adequately introduced to this tool?

Yes ☐ No ☐

4. You were introduced to a tool called "postponing worries." Do you feel that you were adequately introduced to this tool?

Yes ☐ No ☐

5. Would the second session work as a video consultation or a phone call? You may check both "video consultation" and "phone call."

Video consultation ☐ Phone call ☐ Would only work as an in-person meeting at the clinic ☐

6. If you had been offered the choice of either a video consultation, phone call, or in-person meeting at the clinic for the 2nd consultation, which option would you have chosen?

Video consultation ☐ Phone call ☐ In-person meeting at the clinic ☐

The following questions pertain to self-examination your skin:

1. How confident are you in checking your own skin?

Not confident at all ☐ Partly confident ☐ Confident ☐ Very confident ☐

The following questions pertain to the tools and resources you received from the nurse:

1. How useful is the information about the ABCDE rule for you?

Not useful at all ☐ Partly useful ☐ Useful ☐ Very useful ☐

2. How useful is the education in skin self-examination for you?

Not useful at all ☐ Partly useful ☐ Useful ☐ Very useful ☐

3. How useful is the "detached mindfulness" tool for you?

Not useful at all ☐ Partly useful ☐ Useful ☐ Very useful ☐

4. How useful is the "postponing worries" tool for you?

Not useful at all ☐ Partly useful ☐ Useful ☐ Very useful ☐

The following questions pertain to the provided information material "Melanoma - Questions and Answers":

1. How thoroughly have you read "Melanoma - Questions and Answers"?

Have not read it at all ☐ Read less than half ☐

Read more than half ☐ Read the whole thing ☐

The following questions pertain to the satisfaction and usefulness of the follow-up program:

1. Overall, how satisfied are you with this new follow-up method after your melanoma treatment?

Not satisfied at all ☐ Partly satisfied ☐ Satisfied ☐ Very satisfied ☐

2. How much do you agree with the following statements:

By participating in this follow-up program, I have learned:

a) How often I should check my skin

Disagree ☐ Partly agree ☐ Agree ☐

b) How to check my skin

Disagree ☐ Partly agree ☐ Agree ☐

d) To better manage my fear of recurrence or a new case of melanoma

Disagree ☐ Partly agree ☐ Agree ☐

e) To use strategies like "observing mindfulness" or "postponing worries"

Disagree ☐ Partly agree ☐ Agree ☐

f) Where I can find information about additional support for myself or my family

Disagree ☐ Partly agree ☐ Agree ☐

The following questions pertain to whether you would be willing to participate in a research project about the new follow-up program for melanoma. The project would involve the same components you have experienced during the 2 consultations with the nurse. Additionally, it would include 3 more consultations with the nurse up to 2 years after the diagnosis, where the principles of self-monitoring, the ABCDE rule, and tools for managing a cancer diagnosis are repeated. There would also be check-ups with a doctor according to each patient's needs.

Would you say yes to participating in a project about a new follow-up program for melanoma?

Yes ☐ No ☐

If no, why: ____

Appendix 4. Feasibility study participant characteristics (N=14)

Characteristic	n (%)
Mean age (range)	58 (34-82)
Gender	
Men	7 (50)
Women	7 (50)
Education	
Primary education, secondary education or academy profession degree or similar	6 (43)
Bachelor's degree or higher	8 (57)
Employment status	
Working	9 (64)
Retired	5 (36)
Civil status	
Married/cohabitating	10 (71)
Separated/divorced/never married/widowed	4 (29)
Have children	
Yes	12 (86)
No	2 (14)
Yearly household income (before tax EUR)[a]	
53.999 or under	3 (23)
54.000 or more	10 (77)
Melanoma classification, AJCC 8th edition	
Stage IA	9 (64)
Stage IB	5 (36)
Mean months since diagnosis (range)	4 (2.7-4.7)

[a]One did not want to answer this question
